# Intravesical Instillation of Norketamine, a Ketamine Metabolite, and Induced Bladder Functional Changes in Rats

**DOI:** 10.3390/toxics9070154

**Published:** 2021-06-30

**Authors:** Chung-Hsin Yeh, Bo-He Chen, Xiao-Wen Tseng, Chun-Hou Liao, Wei-Kung Tsai, Han-Sun Chiang, Yi-No Wu

**Affiliations:** 1 School of Medicine, College of Medicine, Fu Jen Catholic University, New Taipei City 242, Taiwan; M000732@ms.skh.org.tw (C.-H.Y.); liaoch22@gmail.com (C.-H.L.); 2 Division of Urology, Department of Surgery, Shin Kong Wu Ho-Su Memorial Hospital, Taipei City 111, Taiwan; 3 Graduate Institute of Biomedical and Pharmaceutical Science, Fu Jen Catholic University, New Taipei City 242, Taiwan; s9122099s@hotmail.com; 4 Program in Pharmaceutical Biotechnology, College of Medicine, Fu Jen Catholic University, New Taipei City 242, Taiwan; alitsen@yahoo.com.tw; 5 Division of Urology, Department of Surgery, Cardinal Tien Hospital, New Taipei City 231, Taiwan; 6 Department of Urology, Mackay Memorial Hospital, Taipei City 104, Taiwan; weiko11@gmail.com; 7 Ph.D. Program in Nutrition and Food Science, Graduate Institute of Biomedical and Pharmaceutical Science, Fu Jen Catholic University, New Taipei City 242, Taiwan; 8 Department of Medicine, Mackay Medical College, New Taipei City 252, Taiwan; 9 Mackay Junior College of Medicine, Nursing, and Management, Taipei City 252, Taiwan; 10 Department of Urology, Fu Jen Catholic University Hospital, New Taipei City 243, Taiwan

**Keywords:** intravesical instillation, ketamine cystitis, norketamine, bladder dysfunction

## Abstract

This study aimed to determine the mechanism of ketamine-induced cystitis without metabolism. A total of 24 adult male Sprague-Dawley rats were separated into control, ketamine, and norketamine groups. To induce cystitis, rats in the ketamine and norketamine groups were treated with intravesical instillation of ketamine and norketamine by mini-osmotic pump, which was placed in subcutaneous space, daily for 24 h for 4 weeks. After 4 weeks, all rats were subjected to bladder functional tests. The bladders were collected for histological and pathological evaluation. Compared to control, ketamine treatment demonstrated an increase in the bladder weight, high bladder/body coefficient, contractive pressure, voiding volume, collagen deposition, reduced smooth muscle content, damaged glycosaminoglycan layer, and low bladder compliance. Compared to ketamine, norketamine treatment showed more severe collagen deposition, smooth muscle loss, damaged glycosaminoglycan layer, and increased residual urine. Intravesical administration of ketamine and norketamine induced cystitis with different urodynamic characteristics. Norketamine treatment caused more severe bladder dysfunction than ketamine treatment. Direct treatment of the bladder with norketamine induced symptoms more consistent with those of bladder outlet obstruction than ketamine cystitis. Detailed studies of cellular mechanisms are required to determine the pathogenesis of ketamine cystitis.

## 1. Introduction

Ketamine is a derivative of phencyclidine (PCP) and easily absorbed in humans. It is characterized as an *N*-methyl-d-aspartic acid receptor (NMDAR) antagonist and is a useful analgesic and anesthetic discovered in 1956 and approved by the FDA in 1970 for the use in humans as an anesthetic [1,2,3,4,5]. Lately, ketamine has also been used as an anti-depressant and for the treatment of refractory status epilepticus [4,6]. Recently, ketamine has been used as a recreational and dissociative drug in places including the USA, Europe, and Asia [4,7]. Ketamine abuse can induce ulcerative cystitis without bacterial infection. Clinical side effects involve lower urinary tract dysfunction, including high urination frequency and urgency, bladder pain, and occasional hematuria. In addition, other side effects include low bladder capacity, decreased bladder compliance, higher contractive pressure, and bladder overactivity, as determined in previous studies via urodynamic tests [8,9,10,11,12,13]. The severity of urinary symptoms depends on the frequency and dose of ketamine [14,15]. Because the pathogenesis of ketamine-induced cystitis is still unclear, medical treatment is based on resolving or reducing the symptoms of pain or inflammation, including oral medication, hyperbaric-oxygen therapy, cystoscopic hydrodistention, intravesical medicine injection, or instillation (such as hyaluronic acid, anticholinergic agents, non-steroid or steroid anti-inflammatory drugs, and botulinum toxin A) [11,12,15,16,17,18]. To cure ketamine-induced cystitis, determining the pathophysiology and etiology of the disease is important.

Ketamine and its metabolites are eliminated via the kidney, and the known metabolites include norketamine, dehydronorketamine, hydroxyketamine, and hydroxynorketamine [19]. Among these, the focused metabolite of ketamine in urine is norketamine, which retains an approximately 1/3 to 1/2 anesthetization efficacy of ketamine [10,20,21,22,23,24]. In clinical settings, the concentrations of ketamine and norketamine vary, and the ketamine/norketamine ratio ranges from 0.15 to 2.55 [19,21]. To summarize, the norketamine content in urine is significantly higher than that in ketamine. Thus, several hypotheses underlying the pathogenesis of ketamine-induced cystitis are known which are based on ketamine and norketamine that induce urinary tract dysfunction through different pathways [8,16,25,26,27,28]. One possible mechanism suggests that ketamine and norketamine in urine may directly break the mucosa of the lower urinary tract. During the bladder filling period, severe irritation of the bladder may be observed due to the simultaneous accumulation of ketamine and norketamine [8,16]. Furthermore, ketamine and norketamine penetrate the bladder wall [9,29]. These symptoms were observed in a mouse model of ketamine-induced cystitis. A previous study performed in mice demonstrated the degeneration of muscles and epithelium, increased fibrosis in the lamina propria, fibrosis in the muscular layer, and ketamine-induced cystitis [8,26,30]. However, contradictory results have been reported. In some studies, the results did not demonstrate the symptoms of bladder ulceration or a damaged epithelial layer following ketamine cystitis in humans or mice models [8,31]. These observations indicate that the exact cause of ketamine-induced cystitis remains elusive.

A literature review of ketamine-induced cystitis indicated that all previous studies examined the effects of metabolic ketamine in organisms, however, evidence of the direct effects of ketamine or norketamine on the bladder without considering metabolism is still lacking. In addition, there is no evidence of the individual effects of ketamine or norketamine on the bladder. In this study, we first established a novel rat model with the intravesical instillation of drugs. We aimed to determine the histological and the bladder functional changes induced by the intravesical instillation of ketamine or norketamine. This is the first study to investigate the response of the bladder to ketamine and norketamine without considering metabolism and to determine the individual effects of ketamine-induced cystitis.

## 2. Materials and Methods

### 2.1. Study Design

The experiment was performed on 8-week-old adult Sprague-Dawley (SD) rats. A total of 24 SD rats were randomly divided into control, ketamine, and norketamine groups. Rats in the ketamine group were treated with the intravesical instillation of ketamine hydrochloride (100 mg/mL) 24 h daily for 4 weeks; the norketamine group was treated with the intravesical instillation of norketamine hydrochloride (10 mg/mL) for 24 h daily for 4 weeks, and the control group was not administered with any treatments. After 4 weeks of treatment, bladder function was detected in all rats using the cystometrogram (or cystometry, CMG) test, and then the rats’ bladders were collected and embedded in paraffin blocks for histological and pathophysiological analysis. Body weight and bladder weight were recorded.

### 2.2. Experimental Animal

In this study, male SD rats (8 weeks old) (BioLasco Taiwan Co., Ltd., Taipei, Taiwan) were used, and the study protocols and methods were conducted according to the guidelines of the Declaration of Helsinki, and approved by the Fu Jen Catholic University Institutional Animal Care and Use Committee (approval No.: A10817) on 3 September 2019. All rats were housed in a standard cage at 25 °C under a 12-h light/12-h dark cycle with sufficient food and water.

### 2.3. Intravesical Instillation of Ketamine and Norketamine

Ketamine and norketamine were administered via intravesical instillation. The instillator, Alzet mini-osmotic pump (Alzet, Cupertino, CA, USA), was placed into the rat via surgery according to a previously described procedure [32]. Briefly, Zoletil-50 (1 mL/kg) (Virbac, Carros, France) was intraperitoneally injected for the anesthetization of the rats. A midline abdominal incision was made, and the rat bladder was exposed. A small incision was made in the bladder dome, and a polyethylene micro-tubing 50 (PE50) with a small cuff at the end was placed into the bladder. Then, the catheter and bladder were tied together with a 6–0 polypropylene suture (non-absorbable) (Prolene, Cornelia, GA, USA). The other end of the PE50 catheter was connected to the instillator, which was filled with ketamine hydrochloride (Imalgene 1000, Merial, Lyon, France) (100 mg/mL) or norketamine hydrochloride (Tocris Bioscience, Bristol, UK) (10 mg/mL), and subsequently placed in the subcutaneous space. The abdominal incision was closed with a 6–0 non-absorbable polypropylene suture (Prolene, Cornelia, GA, USA). The rats were then transferred back to the cage. Ketamine and norketamine were administered at a rate of 0.25 µL/h for 4 weeks. The dosages of ketamine hydrochloride and norketamine hydrochloride were based on our previous study, which determined the concentration of ketamine and norketamine in urine after the intraperitoneal injection of ketamine for 4 weeks.

### 2.4. CMG Test

After treatment for 4 weeks, all of the rats underwent bladder functional tests using CMG. The rats were first numbed by isoflurane and exposed to a subcutaneous instillator. The polyethylene micro-tubing 90 (PE90) was substituted for the instillator, and the other end of PE90 was connected to an injector that could fill the bladder with saline at a rate of 0.1 mL/min. The infusion rate was set based on our pre-experiment results of different infusion rates in the CMG analysis, which indicated that the infusion rate can increase the frequency of urination without affecting the normal function of the bladder. The test was performed on awake rats who were placed in a holder, and real-time voiding responses were recorded using an MP36 pressure transducer (Biopac Systems Inc., Santa Barbara, CA, USA) and computer installed recording software, Biopac Student Lab 4.1 (Biopac Systems Inc., Santa Barbara, CA, USA). The micturition parameters of CMG included ICI, threshold pressure, peak pressure, MVV, residual urine volume (infused volume minus voiding volume), and bladder compliance. The threshold pressure is the initial pressure of micturition, and the peak pressure is the maximum pressure of micturition. ICI represents the period between two micturitions. Bladder compliance is the accommodated capability of the bladder (infused volume/delta pressure).

### 2.5. Hematoxylin and Eosin (H&E) Staining, Masson’s Trichrome Staining, and Evaluation of Smooth Muscles and Collagen

After euthanization with an overdose of pentobarbital sodium solution by IP injection, the rat bladders were collected and fixed with 10% formaldehyde (w/v) for 24 h. Then, the tissues were dehydrated, post-fixed, and embedded in paraffin blocks. Before staining, the embedded tissues were sliced into 5-μm thick serial sections, and deparaffinized sections were subjected to hydration by 100%, 95%, 80%, 70% alcohol, and ddH_2_O (each for 5 min). Tissue segments were prepared for H and E staining and Masson’s trichrome staining. In the results of Masson’s trichrome staining, the smooth muscles and collagen are presented in red and blue, respectively. The areas of smooth muscle and collagen were analyzed using the software Scandium 5.2 (Olympus Soft Imaging Solutions GmbH, Muenster, Germany). The ratio of collagen to smooth muscle was indicated by the fibrosis levels of smooth muscle, and the ratio of smooth muscle to tissue area was indicated as the muscle content of the bladder depicted in percentage.

### 2.6. Statistical Analysis

Data are presented as means ± standard deviations, and the Scheffe post hoc test was used for statistical analysis. Statistical analyses were performed using SPSS v.18.0 (SPSS Inc., Chicago, IL, USA), and statistical significance was set at *p* < 0.05.

## 3. Results

### 3.1. Intravesical Instillation of Ketamine or Norketamine for Four Weeks Altered the Body Weight and Bladder Weight of Rats

**Figure 1:**
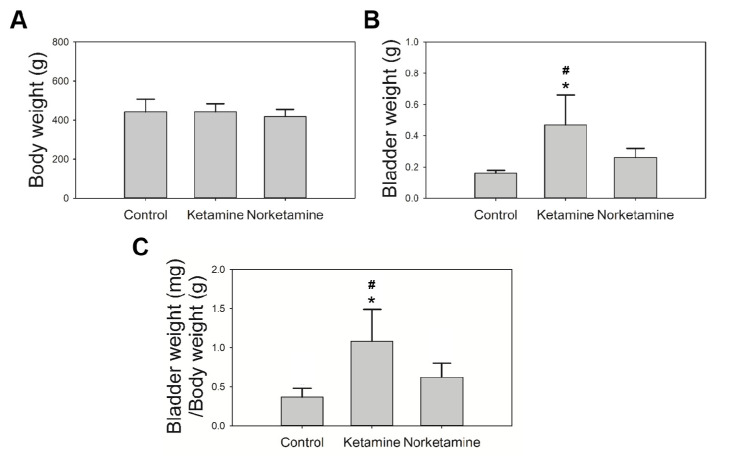
The body weight and bladder weight of rats in each group. The body weight (**A**) was recorded after administering the treatment for 4 weeks; the bladder weight (**B**) was recorded after euthanization; the bladder/body coefficient was calculated (**C**). * indicates statistical differences in comparison with the control group, *p* < 0.05; ^#^ indicates statistical differences in comparison with the norketamine group, *p* < 0.05.

The body weight and bladder weight of rats in this study were recorded (Figure 1). The body weights of the rats did not differ significantly after the intravesical instillation treatment for 4 weeks between the groups. In contrast, the bladder weight of the rats in the ketamine group was significantly heavier than that of the rats in the norketamine group (*p* = 0.031) and control group (*p* = 0.002). However, the bladder weight of the rats in the norketamine group was higher than that of the rats in the control group but the difference was not statistically significant (*p* = 0.357). The bladder weight/body weight ratio, as well as the bladder weight alone, can demonstrate more objective differences. The results indicated that the intravesical instillation of ketamine can induce severe changes in bladder ratio compared to that of norketamine.

### 3.2. Intravesical Instillation of Ketamine or Norketamine Impaired the Bladder Function

**Figure 2:**
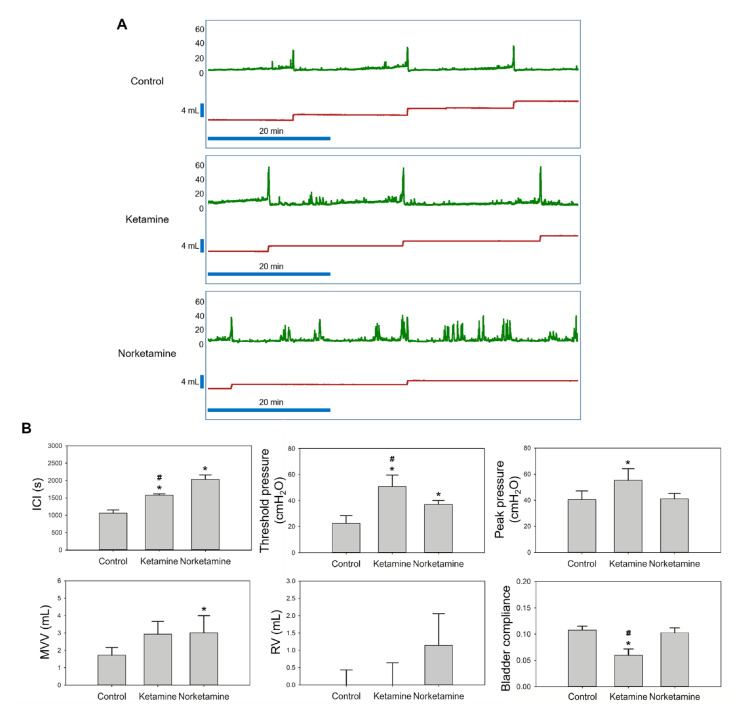
The cystometric analysis (CMG) of control, ketamine-treated, and norketamine-treated rats. (**A**) A one-hour period of CMG in each group observed with the recording software, Biopac Student Lab 4.1 (Biopac Systems Inc., Santa Barbara, CA, USA); (**B**) Statistical analysis of the intercontraction intervals (ICI), threshold pressure, peak pressure, residual urine volume (RV), mean voiding volume (MVV), and bladder compliance. * indicates statistical differences in comparison with the control group, *p* < 0.05; ^#^ indicates statistical differences in comparison with the norketamine group, *p* < 0.05.

The results of the bladder functional test are shown in Figure 2A, which captured a one-hour period of the cystometrogram (CMG) test. The results observed the hyperactive contraction of the treatment groups. At the same time, the other parameters of CMG are also presented in Figure 2B. In the ketamine group, CMG test results showed significantly increased intercontraction intervals (ICI), threshold pressure, peak pressure, and significantly decreased bladder compliance compared to the control group. These findings indicate that the major effects of ketamine include strong voiding pressure and low bladder compliance. In contrast, the norketamine group showed the longest ICI and the highest mean voiding volume (MVV) of all groups, however, no significant differences were observed in voiding pressure compared to that of the control group (*p* = 0.934). In summary, the major characteristic of the norketamine group involved the absence of enough pressure to empty the bladder. We observed that the residual urine was approximately 0.91 mL. Specifically, the norketamine showed detrusor underactivity. Based on the aforementioned results, both the intravesical instillations of ketamine and norketamine impaired bladder function.

### 3.3. Histological Changes Corresponding to Ketamine and Norketamine-Induced Damage in Bladder

**Figure 3:**
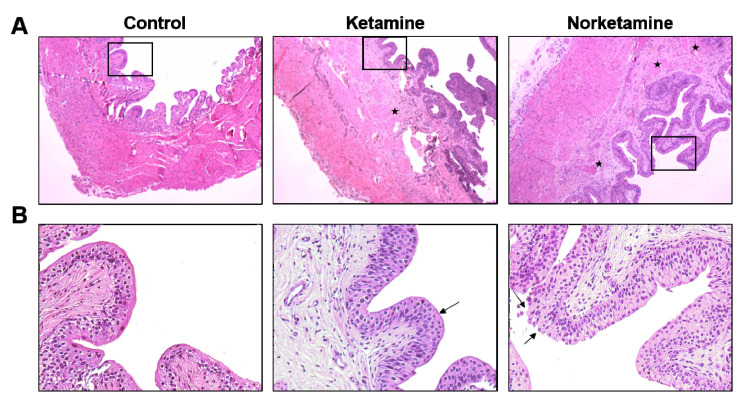
Haematoxylin and eosin staining of the bladder from each group. The images of the upper row (**A**) were taken at 40× magnification and images of the lower row (**B**) were taken at 200× magnification. The star marks indicate the edema sites in the lamina propria, and the damaged glycosaminoglycan (GAG) layer shows in the edge of the epithelial layer (arrows).

The histological characteristics of the bladder were evaluated by hematoxylin and eosin (H and E) staining and Masson’s trichrome staining. The results of H and E staining (Figure 3) demonstrated a thicker epithelial layer and lamina propria in the bladder tissues of the rats of the ketamine and norketamine groups compared to those of the rats in the control group. Meanwhile, the glycosaminoglycan (GAG) layer was damaged, and an unusual proliferation of epithelium was observed in the treatment groups.

**Figure 4:**
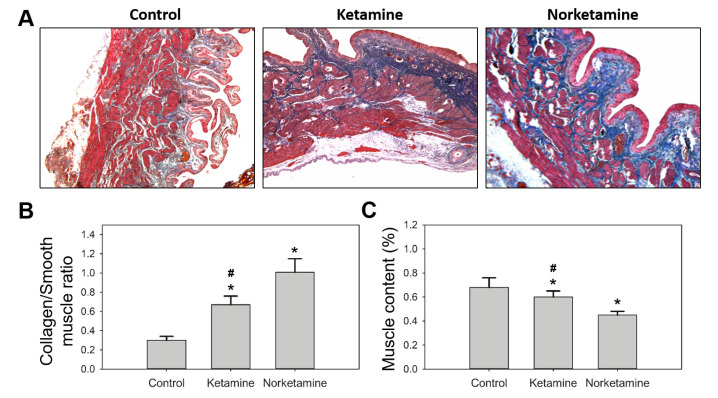
Masson’s trichrome staining of the bladder of the rats in each group (**A**), and the statistical analysis of collagen/smooth muscle (**B**) and smooth muscle content (%) of each group (**C**). * indicates statistical differences in comparison with the control group, *p* < 0.05; ^#^ indicates statistical differences in comparison with the norketamine group, *p* < 0.05.

Simultaneously, the edema of the lamina propria was observed in the treatment groups, especially in the norketamine group. The results of Masson’s trichrome staining (Figure 4) demonstrated increased collagen deposition (blue color) in the bladder tissue of the treatment groups along with a thicker epithelial layer. In the statistical analysis, the collagen/smooth muscle ratio was significantly increased for the treatment groups. The highest value was observed in the norketamine group, indicating severe fibrosis of the bladder. Simultaneously, the smooth muscle content was significantly decreased after treatment with ketamine (*p* = 0.000) or norketamine (*p* = 0.012) in comparison with that observed in the control group.

## 4. Discussion

In clinical practice, a reduction in the body weight of ketamine abusers is a common symptom [28,33,34]. However, in this study, following four weeks of treatment, the body weights of the rats administered with ketamine were not significantly different from those belonging to the control. This is similar to a previous study in which ketamine was administered to the rats via an intraperitoneal injection for 12 weeks. The results demonstrated that the body weight reduced significantly until the 12th week [34]. The differences in the weight between the control and the ketamine-treated mice increased with longer periods of treatment and higher dosage [14]. Weight loss is based on the side effects of ketamine, such as appetite loss and vomiting [24,35]. In contrast, body weight, bladder weight, and bladder/body coefficient of rats in the ketamine group were increased. This is a characteristic of ketamine cystitis in rats, as demonstrated in previous studies [18,34]. We suggest that the size and weight of the bladder increase with ketamine intake but not with norketamine.

The symptoms of ketamine-induced dysfunction include increased frequency, urgency, smaller bladder capacity, decreased bladder compliance, high contractive pressure, and hyperactive bladder [8,9,10,11,12,13]. The severity of urinary symptoms is directly related to the dosage and frequency of ketamine intake [14]. Our results showed that the symptoms of bladder hyperactivity, high contractive pressure, and decreased bladder compliance were consistent with the clinical findings. The results were also consistent with those observed with other rat models that were treated with ketamine by IP injection [10,13,36]. A recent study indicated that strong contractive pressure was correlated with transient receptor potential cation channel subfamily V (TRPV) proteins in humans. The results showed that the expression of TRPV4 was significantly increased in the epithelium of patients with severe ketamine cystitis [12]. When TRPV4 is overexpressed in the bladder, calcium concentration is increased, which leads to an increase in the frequency of bladder contraction [12,37]. Nevertheless, the evidence for this is still lacking and needs further confirmation.

In contrast, some symptoms of rats with direct norketamine-treatment were not consistent with those observed with the other rat models, such as increased residual urine and voiding volume. These were consistent with the symptoms of bladder outlet obstruction (BOO), a common lower urological chronic condition. The urodynamic test characterization of BOO demonstrates not only increased pressure during urination, but also increased bladder capacity and incomplete emptying of the bladder [38,39,40,41]. Several studies have shown that BOO is involved in the pathogenesis of bladder dysfunction and results from numerous diseases, including benign prostatic obstruction, bladder neck obstruction, posterior urethral valves, and urethral strictures [40,42]. In recent decades, a three-stage model has been proposed as a hypothesis for bladder dysfunction. This hypothesis is based on the remodeling of the bladder muscle (detrusor), involving the hypertrophy phase, compensation phase, and decompensation phase [40,43,44]. In detail, increased oxidative stress, inflammatory response, and ischemia result in urinary dysfunction, and detrusor hypertrophy results as a consequence of high ureteral resistance. Hypertrophy alters the expression of muscle proteins. Many studies have proved that smooth muscles are substituted by collagen in humans, however, the cause has not yet been demonstrated. This condition involves an increase in the mass, weight, wall thickness of the bladder, and degeneration of the detrusor [40,45,46,47]. The characteristics of the compensation phase involve increased contraction during the voiding phase and over-activation of the detrusor during the filling phase. The decompensation phase is present during detrusor underactivity, which is characterized by low detrusor wall thickness, large bladder capacity, and muscle fibrosis. Interestingly, patients with detrusor underactivity show low expression of inducible nitric oxide synthase and E-cadherin than patients with detrusor over-activation [40,41]. A clinical study demonstrated that the collagen content of the bladder in diabetes patients was remarkably higher than that of non-diabetic patients. However, diabetes is also related to detrusor underactivity [47]. According to this hypothesis, the rats in the ketamine group could be at the compensation phase of bladder dysfunction, based on higher voiding pressure, and the rats in the norketamine group could be at the decompensation phase of bladder dysfunction, based on the indistinguishable voiding pressure compared to the control rats, incomplete emptying of the bladder, and increased bladder capacity.

Compared with normal humans in terms of histological symptoms, denudation of the epithelium is often observed in cystoscopy under clinical settings, however, only about 50% of patients demonstrate the symptoms [8,16,48]. In our results, a thinner GAG layer and increased epithelial proliferation, but without denudation, were observed in both treatment groups. The identification is consistent with a previous study, which confirmed the intact umbrella cell apical membranes by electron microscopy analysis. At the same time, the results of immunofluorescence staining also demonstrated a high expression of uroplakin (a specific protein of urothelium) in umbrella cells [31]. The observation combined with the results of CMG indicated that the damaged epithelium was not the only or direct influence of urinary dysfunction. 

## 5. Conclusions

The study demonstrated that the intravesical administration of ketamine and norketamine induced cystitis with different urodynamic characteristics and pathological changes. Norketamine treatment seemed to result in more severe bladder dysfunction than ketamine treatment. However, these results need more research evidence to prove the potential mechanism of occurrence. We also demonstrated that a direct treatment of the bladder with norketamine induced complex symptoms, more consistent with those observed following the obstruction of the bladder outlet than ketamine cystitis. However, detailed studies of cellular mechanisms are still imperative to elucidate the pathogenesis of ketamine cystitis in the future.

## References

[1] Domino E.F., Chodoff P., Corssen G. (1965). Pharmacologic effects of CI-581, a new dissociative anesthetic, in man. Clin. Pharmacol. Ther..

[2] Grant I.S., Nimmo W.S., Clements J.A., Wyant G.M. (1982). Pharmacokinetics and Analgesic Effects of I.M. and Oral Ketamine. Surv. Anesthesiol..

[3] Dong T.T., Mellin-Olsen J., Gelb A.W. (2015). Ketamine: A growing global health-care need. Br. J. Anaesth..

[4] Wei Y., Chang L., Hashimoto K. (2020). A historical review of antidepressant effects of ketamine and its enantiomers. Pharmacol. Biochem. Behav..

[5] Lichtwardt J.R., Girgis S. (1985). Transurethral resection of prostate with intravenous sedation. Urology.

[6] Rosati A., De Masi S., Guerrini R. (2018). Ketamine for Refractory Status Epilepticus: A Systematic Review. CNS Drugs.

[7] Chang C.-M., Wu T.L., Ting T.-T., Chen C.-Y., Su L.-W., Chen W.J. (2019). Mis-anaesthetized society: Expectancies and recreational use of ketamine in Taiwan. BMC Public Health.

[8] Chu P.S.-K., Ma W.-K., Wong S.C.-W., Chu R.W.-H., Cheng C.-H., Wong S., Tse J.M.-L., Lau F.-L., Yiu M.-K., Man C.-W. (2008). The destruction of the lower urinary tract by ketamine abuse: A new syndrome?. BJU Int..

[9] Tsai T.-H., Cha T.-L., Lin C.-M., Tsao C.-W., Tang S.-H., Chuang F.-P., Wu S.-T., Sun G.-H., Yu D.-S., Chang S.-Y. (2009). Ketamine-associated bladder dysfunction. Int. J. Urol..

[10] Chuang S.-M., Liu K.-M., Li Y.-L., Jang M.-Y., Lee H.-H., Wu W.J., Chang W.-C., Levin R.M., Juan Y.-S. (2013). Dual involvements of cyclooxygenase and nitric oxide synthase expressions in ketamine-induced ulcerative cystitis in rat bladder. Neurourol. Urodynamics.

[11] Sihra N., Ockrim J., Wood D. (2018). The effects of recreational ketamine cystitis on urinary tract reconstruction—A surgical chal-lenge. BJU Int..

[12] Yang H.H., Jhang J.F., Hsu Y.H., Jiang Y.H., Zhai W.J., Kuo H.C. (2021). Smaller bladder capacity and stronger bladder contrac-tility in patients with ketamine cystitis are associated with elevated TRPV1 and TRPV4. Sci. Rep..

[13] Wang J., Chen Y., Gu D., Zhang G., Chen J., Zhao J., Wu P. (2017). Ketamine-induced bladder fibrosis involves epithelial-to-mesenchymal transition mediated by transforming growth factor-β1. Am. J. Physiol. Physiol..

[14] Winstock A.R., Mitcheson L., Gillatt D.A., Cottrell A.M. (2012). The prevalence and natural history of urinary symptoms among recreational ketamine users. BJU Int..

[15] Li C.-C., Wu S.-T., Cha T.-L., Sun G.-H., Yu D.-S., Meng E. (2019). A survey for ketamine abuse and its relation to the lower urinary tract symptoms in Taiwan. Sci. Rep..

[16] Shahani R., Streutker C., Dickson B., Stewart R.J. (2007). Ketamine-Associated Ulcerative Cystitis: A New Clinical Entity. Urology.

[17] Jhang J.-F., Hsu Y.-H., Kuo H.-C. (2015). Possible pathophysiology of ketamine-related cystitis and associated treatment strategies. Int. J. Urol..

[18] Lee Y.-L., Lin K.-L., Chuang S.-M., Lee Y.-C., Lu M.-C., Wu B.-N., Wu W.-J., Yuan S.-S.F., Ho W.-T., Juan Y.-S. (2017). Elucidating Mechanisms of Bladder Repair after Hyaluronan Instillation in Ketamine-Induced Ulcerative Cystitis in Animal Model. Am. J. Pathol..

[19] Moore K.A., Sklerov J., Levine B., Jacobs A.J. (2001). Urine concentrations of ketamine and norketamine following illegal consumption. J. Anal. Toxicol..

[20] Mozayani A. (2002). Katamine—Effects on Human Performance and Behavior. Forensic Sci. Rev..

[21] Wang K.-C., Shih T.-S., Cheng S.-G. (2005). Use of SPE and LC/TIS/MS/MS for rapid detection and quantitation of ketamine and its metabolite, norketamine, in urine. Forensic Sci. Int..

[22] Wu G.-J., Chan H., Lee M.-R., Chen C.-Y., Yang D.-Y., Cheng F.-C. (2007). Simultaneous measurement of urinary ketamine, nor-ketamine, and dehydronorketamine by liquid chromatography-atmospheric pressure chemical ionization mass spectrometry. J. Chin. Chem. Soc..

[23] Kim E.-M., Lee J.-S., Choi S.-K., Lim M.-A., Chung H.-S. (2008). Analysis of ketamine and norketamine in urine by automatic solid-phase extraction (SPE) and positive ion chemical ionization–gas chromatography–mass spectrometry (PCI–GC–MS). Forensic Sci. Int..

[24] Zanos P., Moaddel R., Morris P.J., Riggs L.M., Highland J.N., Georgiou P., Pereira E.F.R., Albuquerque E.X., Thomas C.J., Gould T.D. (2018). Ketamine and Ketamine Metabolite Pharmacology: Insights into Therapeutic Mechanisms. Pharmacol. Rev..

[25] Lin C.-C., Lin A.T.-L., Yang A.-H., Chen K.-K. (2016). Microvascular Injury in Ketamine-Induced Bladder Dysfunction. PLoS ONE.

[26] Gu D., Huang J., Yin Y., Shan Z., Zheng S., Wu P. (2014). Long-term ketamine abuse induces cystitis in rats by impairing the bladder epithelial barrier. Mol. Biol. Rep..

[27] Wei Y., Yang J.R., Yin Z., Guo Q., Liang B.L., Zhou K.Q. (2013). Genitourinary toxicity of ketamine. Hong Kong Med. J..

[28] Chen H., Vandorpe D.H., Xie X., Alper S.L., Zeidel M.L., Yu W. (2020). Disruption of Cav1.2-mediated signaling is a pathway for ketamine-induced pathology. Nat. Commun..

[29] Riedl C.R., Engelhardt P.F., Daha K.L., Morakis N., Pflüger H. (2008). Hyaluronan treatment of interstitial cystitis/painful bladder syndrome. Int. Urogynecol. J. Pelvic Floor Dysfunct..

[30] Tan S., Chan W., Wai M.S., Hui L.K., Hui V.W., James A.E., Yeung L., Yew D. (2011). Ketamine effects on the urogenital system-changes in the urinary bladder and sperm motility. Microsc. Res. Tech..

[31] Rajandram R., Ong T.A., Razack A.H.A., Maciver B., Zeidel M., Yu W. (2016). Intact urothelial barrier function in a mouse model of ketamine-induced voiding dysfunction. Am. J. Physiol. Physiol..

[32] Uvin P., Everaerts W., Pinto S., Alpizar Y.A., Boudes M., Gevaert T., Voets T., Nilius B., Talavera K., De Ridder D. (2012). The Use of Cystometry in Small Rodents: A Study of Bladder Chemosensation. J. Vis. Exp..

[33] Chu P.S., Kwok S.C., Lam K.M., Chu T.Y., Chan S.W., Man C.W., Ma W.K., Chui K.L., Tse M.L., Lau F.L. (2007). ‘Street ketamine’-associated bladder dysfunction: A report of ten cases. Hong Kong Med. J..

[34] Wu Z.-G., Chen F., Wu H., Chen J.-X., Wei Q.-T., Fu Y.-Q., Lu X., Ye Y., Yan Y.-Y., Liao L.-C. (2018). Urinary metabonomics of rats with ketamine-induced cystitis using GC-MS spectroscopy. Int. J. Clin. Exp. Pathol..

[35] Shen C.-H., Wang S.-T., Wang S.-C., Lin S.-M., Lin L.-C., Dai Y.C., Liu Y.W. (2019). Ketamine-induced bladder dysfunction is associated with extracellular matrix ac-cumulation and impairment of calcium signaling in a mouse model. Mol. Med. Rep..

[36] Kim A., Yu H.Y., Heo J., Song M., Shin J.-H., Lim J., Yoon S.-J., Kim Y., Lee S., Kim S.W. (2016). Mesenchymal stem cells protect against the tissue fibrosis of ketamine-induced cystitis in rat bladder. Sci. Rep..

[37] Mochizuki T., Sokabe T., Araki I., Fujishita K., Shibasaki K., Uchida K., Naruse K., Koizumi S., Takeda M., Tominaga M. (2009). The TRPV4 Cation Channel Mediates Stretchevoked Ca^2+^ Influx and ATP Release in Primary Urothelial Cell Cultures. J. Biol. Chem..

[38] Dmochowski R.R. (2005). Bladder Outlet Obstruction: Etiology and Evaluation. Rev. Urol..

[39] Metcalfe P.D., Wang J., Jiao H., Huang Y., Hori K., Moore R.B., Tredget E.E. (2010). Bladder outlet obstruction: Progression from inflammation to fibrosis. BJU Int..

[40] Bosch R., Abrams P., Averbeck M.A., Agró E.F., Gammie A., Marcelissen T., Solomon E. (2019). Do functional changes occur in the bladder due to bladder outlet obstruction?—ICI-RS 2018. Neurourol. Urodyn..

[41] Fusco F., Creta M., De Nunzio C., Iacovelli V., Mangiapia F., Marzi V.L., Agrò E.F. (2018). Progressive bladder remodeling due to bladder outlet obstruction: A systematic review of morphological and molecular evidences in humans. BMC Urol..

[42] Al-Hayek S., Thomas A., Abrams P. (2004). Natural history of detrusor contractility—minimum ten-year urodynamic follow-up in men with bladder outlet obstruction and those with detrusor. Scand. J. Urol. Nephrol. Suppl..

[43] Yang X., Wang J., Wang R., Xu Y., Chen F., Tang L., Ren W., Fu L., Tan B., Huang P. (2019). Time-dependent functional, morphological, and molecular changes in diabetic bladder dysfunction in streptozotocin-induced diabetic mice. Neurourol. Urodyn..

[44] Daneshgari F., Liu G., Birder L., Hanna-Mitchell A.T., Chacko S. (2009). Diabetic Bladder Dysfunction: Current Translational Knowledge. J. Urol..

[45] Mannikarottu A.S., Hypolite J.A., Zderic S.A., Wein A.J., Chacko S., Disanto M.E. (2005). Regional alterations in the expression of smooth muscle myosin isoforms in response to partial bladder outlet obstruction. J. Urol..

[46] Mirone V., Imbimbo C., Sessa G., Palmieri A., Longo N., Granata A.M., Fusco F. (2004). Correlation between detrusor collagen content and urinary symptoms in patients with prostatic obstruction. J. Urol..

[47] Averbeck M.A., De Lima N.G., Motta G.A., Beltrao L.F., Filho N.J.A., Rigotti C.P., Dos Santos W.N., Dos Santos S.K., Da Silva L.F., Rhoden E.L. (2018). Collagen content in the bladder of men with LUTS undergoing open prostatectomy: A pilot study. Neurourol. Urodynamics.

[48] Lin H.-C., Lee H.-S., Chiueh T.-S., Lin Y.-C., Cha T.-L., Meng E. (2015). Histopathological assessment of inflammation and expression of inflammatory markers in patients with ketamine-induced cystitis. Mol. Med. Rep..

